# Pelvic floor therapy program for the treatment of female urinary incontinence in Belize: a pilot study

**DOI:** 10.3389/fgwh.2024.1325259

**Published:** 2024-02-09

**Authors:** David E. Rapp, Jacqueline Zillioux, Fionna Sun, Marieke Jones, Michelle Little, Jeanice Mitchell

**Affiliations:** ^1^ Department of Urology, University of Virginia, Charlottesville, VA, United States; ^2^ Global Surgical Expedition, Glen Allen, VA, United States; ^3^ Department of Public Health Sciences, University of Virginia, Charlottesville, VA, United States; ^4^ Women in Motion Physical Therapy, Charlottesville, VA, United States; ^5^ Integrity Rehab and Home Health, Killeen, TX, United States

**Keywords:** pelvic floor therapy, stress urinary incontinence, urge urinary incontinence, quality of life, pelvic floor disorders

## Abstract

**Introduction:**

Urinary incontinence (UI) is highly prevalent in low- and middle-income countries (LMIC). Concurrently, the availability of surgical or conservative UI treatments in LMIC is limited.

**Methods:**

We conducted a prospective feasibility study of Belize women with UI treated with pelvic floor physical therapy (PFPT) and education (PFE). Patients received individual PFPT/PFE over 2 days, consisting of biofeedback-enhanced PFMT in addition to behavioral, dietary, and general pelvic education. Patient completed a daily 6-month home regimen including 7 PFMT exercises (total 70 repetitions) comprising both endurance and quick flick exercises. Patients also performed comprehensive dietary and behavioral modification activities. Outcomes were assessed at baseline and 6-months, including validated symptom (ICIQ-FLUTS) and QOL (IIQ-7) questionnaires, and strength testing (PERFECT score, perineometry).

**Results:**

Twenty-eight patients underwent baseline assessment. Four patients were lost to in-person 6-month follow-up, with two of these patients completing subjective assessment only by telephone. The mean (±SD) patient age, BMI, and parity were 50.0 (±10.0) years, 33.2 (±5.8), and 2.8 (±1.5). Provider assessment demonstrated patient comprehension of basic, endurance, and quick flick pelvic floor contractions in 28 (100%), 24 (86%), and 24 (86%) patients, respectively. At 6-month follow-up, significant improvements were seen across multiple validated questionnaire and strength measurement assessments. Median patient-reported improvement level was 7.0 on a 10-point Likert scale.

**Discussion:**

Study patients demonstrated good understanding of PFMT/PFE and program completion was associated with significant improvements across a variety of subjective incontinence and quality of life outcomes, as well as objective strength testing.

## Introduction

The worldwide impact of surgical disease is vast and estimated to account for approximately one third of the global burden of disease (1, 2). The lack of access to timely, safe, and affordable surgical care causes an estimated 17 million deaths annually, as well as significant years lived with disability and quality of life reduction (1, 2). The 2015 Lancet Commission on Global Surgery (LCGS) estimated that five billion people lack access to safe and affordable surgical care and set forth a variety of proposals to help improve the delivery of surgical care and infrastructure (3).

Female pelvic floor disorders (PFD), including urinary incontinence (UI) and pelvic organ prolapse (POP), are highly prevalent in low- and middle-income countries (LMIC) and have a tremendous negative impact to women (4, 5). Numerous surgical options (e.g., urethral sling placement, vaginal vault suspension, sacrocolpopexy) are highly effective and durable treatments for PFD. However, despite the disability and well-described impact to QOL, mental health, and general functioning, PFD often receive less focus when compared to other surgical diseases and essential surgeries (6, 7).

Global surgical expedition (GSE) is a medical charity that builds surgical infrastructure and provides surgical care internationally to populations in need. As part of prior work focused on better understanding the significant impact of PFD in LMIC, we have previously reported that, in addition to these deleterious impacts to health, PFD significantly also impacts the financial well-being of women. Our data demonstrated that a large proportion of Belizean women report missing work and/or impairment while at work related to PFD, resulting in a loss of per capita income ranging from 7%–9% (8). These findings are consistent with the LCGS report, which emphasizes the economic impact of surgical disease and urges focus on the ability of surgery to not only acheive health but also related benefits including welfare and economic development (3).

Given these broad negative impacts of PFD, much of GSE's efforts have included focus on building access to surgical care for PFD. At the same time, pelvic floor physical therapy (PFMT) and behavioral techniques (i.e., behavioral and dietary modifications) are recognized as a standard treatment for female UI and is supported by a large body of research and expert guideline statements (9, 10). Notably, published investigation demonstrates that completion of PFMT avoids the need for surgery in half of patients with stress urinary incontinence when assessed at 1-year follow-up (11). Accordingly, GSE initiated development of a parallel PFMT program for UI in Belize to support its surgical programming and thereby comprehensively treat UI in women with both surgical and non-surgical approaches. The aim of this study was to assess continence and QOL outcomes in Belizean women treated with PFMT supplemented by a 2-month home exercise program. Secondarily, we aimed to understand program feasibility, including patient exercise comprehension and compliance.

## Materials and methods

We conducted a prospective pilot study of women in Belize with UI treated with pelvic floor physical therapy (PFMT) and education (PFE). Patients were recruited as part of short-term visiting surgical trips overseen by GSE. Trips were conducted between November 2021-January 2023 across different regions in Belize. GSE trips begin with a clinic to evaluate patients with urologic disorders. Women reporting UI were then screened for study participation. Patients with stress (SUI), urge (UUI), or mixed (MUI) urinary incontinence were eligible for participation. Inclusion criteria also included age ≥18 years and ability to understand verbal and written educational materials in English or Spanish. Exclusion criteria included a history of prior anti-incontinence surgery.

Enrolled patients received individual PFMT/PFE by one of two therapists (JM, ML) over the course of 2 days (1 h per day). Pelvic floor training included biofeedback-enhanced PFMT with pelvic floor activation training (dynamic and functional activities). Pelvic floor education consisted of broad education focused on pelvic floor anatomy and function, behavioral modification training, and dietary education. Behavioral modification focuses on behavioral approaches to promote bladder health, including toileting posture and the use of scheduled voiding. Dietary education also promotes bladder control by eliminating foods and drinks that may cause bladder instability or irrigation (e.g., caffeine, acid). A detailed list of specific behavorial and dietary modification techniques used in our study is provided in Supplementary Material S1. Patients received printed education materials to supplement verbal education and detailing the home PFE program (Supplementary Material S1).

Patients were then instructed to complete a daily 6-month home regimen of PFMT exercises. The home PFMT program consisted of seven exercises (10 repetitions/exercise) and included both endurance and quick flick exercises across supine, seated, and standing positions (Supplementary Material S1). In addition, patients performed comprehensive dietary and behavioral modification activities.

### Outcomes, continence and quality of life

Patients outcomes were assessed at baseline and 6-month timepoints, including validated symptom (ICIQ-FLUTS) and QOL (IIQ-7) questionnaires, strength testing (PERFECT score, perineometry), and items assessing PFMT comprehension and subjective improvement (12–14). Primary outcomes included ICIQ-FLUTS domain items for stress urinary incontinence (SDS, stress domain score), urge urinary incontinence (UIDS), as well as PERFECT score domains. The ICIQ-FLUTS is a validated questionnaire with domains focused on lower urinary tract symptoms. ICIQ-FLUTS domains scores ranges from 0 to 4 (0='Never'; 1='Occasionally'; 2='Sometimes'; 3='Most of the time'; 4='All of the time'). The PERFECT score domains assess characteristics of pelvic floor muscle strength and endurance, with higher scores being better.

Secondary outcomes included ICIQ-FLUTS domain items for urgency (UDS) and nocturia (NDS), patient-reported improvement on 10-point Likert scale, IIQ-7 QOL score (lower score being better), and strength measurements by perineometry. Specifically, we assessed the perineometry difference between resting and working maximum pressures during both endurance and quick flick exercises.

### Program comprehension

In addition to comparative assessment detailed above, baseline also included outcomes focused on assessing patient comprehension of PFMT. Following initial training, patients were instructed to complete a basic pelvic floor contraction, followed by endurance (>5 s), and quick flick (multiple 1 s) contractions. Digital assessment was used to assess patients ability and positive comprehension was considered if patients correctly executed these contractions using appropriate muscle groups.

### Outcomes, other

Additional verbal questions were used to assess patient compliance and program feedback. Dichotomous questions (Y/N) assessed whether patients found the program helpful and understood the program. Patient compliance was assessed by verbal interview question to ensure that patients began the home program and had performed pelvic exercises at home.

### Statistical analysis

Descriptive statistics are reported as mean ± standard deviation for continuous variables and *n* (%) for categorical variables. To analyze change from baseline to 6-months post PFMT/PFE we used the Wilcoxon signed rank test with continuity correction. These results are presented with median (IQR). All analyses were conducted using R version 4.1.

## Results

Twenty-eight patients were enrolled and underwent baseline assessment. Four patients were lost to follow-up for in-person assessment at 6 months. However, the study team was able to contact via telephone two of the four patients lost to follow-up and these patients completed subjective assessment (ICIQ-FLUTS, IIQ-7, and improvement/comprehension items). Sample size is noted throughout results presentation.

Patient characteristics are detailed in Table 1. Women had an average age of 50 ± 10 and 2.79 ± 1.50 previous pregnancies (Table 1). Eighteen, five, and five patients had MUI, UUI, and SUI, respectively. On average, patients had experienced urinary incontinence for 79 ± 83 months and none of the women had undergone prior treatment for urinary incontinence, including both pharmaceutical and surgical therapies.

**Table 1:** Patient characteristics.

Characteristic	*N* = 28
Age	50 ± 10
BMI (*N* = 27)	33.2 ± 5.8
Parity	2.79 ± 1.50
Duration of UI (months)	79 ± 83
UI subtype	
MUI UUI UUI	18 (64%) 5 (18%) 5 (18%)
Smoking status	0 (0%)
Prior hysterectomy	4 (14%)
Prior treatment	0 (0%)

UI, urinary incontinence; MUI, mixed urinary incontinence; SUI, stress urinary incontinence; UUI, urge urinary incontinence.

Mean SD, *N* (%).

*N* = 28 except where noted).

Initial comprehension of PFMT was demonstrated by the majority of patients, with comprehension of basic pelvic floor activation, endurance contraction, and quick flick contraction demonstrated in 100%, 86%, and 86% of patients, respectively.

Subjective outcomes are detailed in Table 2. All patients assessed reported compliance with performing home exercises. Following PFMT, significant improvements were seen across all ICIQ domains (*p* < 0.05, all comparisons). Similarly, significant improvement in quality of life was seen, with median IIQ-7 sum improving from 7.5 to 0 in comparison of baseline and 6-months scores (*p* = 0.017). On a scale from 1 to 10, patients reported a median improvement of 7. All patients responded yes to finding the pelvic program helpful and that they understood the program.

**Table 2:** Patient-reported outcomes (*n* = 26).

Characteristic	Baseline^a^	6-months follow-up^a^	*p*-value*
Questionnaire outcomes			
SUI domain (ICIQ)	2.0 (1.3, 3.0)	0.5 (0.0, 2.0)	0.009
UUI domain (ICIQ)	2.0 (1.0, 2.0)	0.0 (0.0, 1.8)	0.007
Urge domain (ICIQ)	2.0 (1.0, 2.0)	1.0 (0.0, 1.8)	0.003
Nocturia domain (ICIQ)	3.0 (2.0, 3.8)	2.0 (1.3, 3.0)	0.026
IIQ sum score	7.5 (2.0, 11.8)	0.0 (0.0, 8.2)	0.017
Patient reported outcomes			
Patient-reported improvement		7.0 (5.0, 8.0)	
Patient-reported understanding		26 (100%)	
Patient-reported helpful		26 (100%)	** **

^a^ Median (IQR); *n* (%).

* Wilcoxon signed rank test with continuity correction.

Objective outcomes are detailed in Table 3. Comparison of baseline and 6-month PERFECT outcomes demonstrated statistically significant improvements across Power, Endurance, and Fast Domains. Improvements were also seen across Repetition domain, however, the increase was not statistically significant (*p* = 0.086). Perineometry assessment failed to demonstrate statistically significant differences across both fast and endurance contractions.

**Table 3:** Objective outcomes (*n* = 24).

Characteristic	Baseline^a^	6-months follow-up^a^	*p*-value*
Perfect			
Power	2.0 (2.0, 2.2)	4.0 (3.0, 4.0)	<0.001
Endurance (*s*)	2.0 (2.0, 4.2)	4.0 (3.0, 6.0)	0.014
Repetitions (*n*)	3.0 (2.0, 4.0)	4.0 (3.0, 5.2)	0.086
Fast (*n*)	8.0 (5.0, 10.0)	16.0 (10.5, 27.0)	0.002
Perineography max change^b^			
Fast max change (mEq)	−2.1 (−4.7, 0.6)	−1.5 (−3.9, 0.0)	0.6
Endurance max change (mEq)	−7.4 (−12.4, −2.3)	−5.2 (−11.1, −1.9)	0.5

^a^ Median (IQR).

^b^ Mean max work—mean max resting.

* Wilcoxon signed rank test with continuity correction; Wilcoxon signed rank exact test.

## Discussion

Our study demonstrates the successful implementation of a PFMT and PFE program in the treatment of UI in Belizean women. Our analysis included several stepwise outcomes important to comprehensively assessing program feasibility. First, our analysis showed that patients were able to understand program education and materials. Based on this education, women correctly performed basic pelvic muscle contractions at program baseline. Second, patients reported a high rate of program compliance at 6-month follow-up. Third, program completion was associated with a variety of subjective and objective improvements, including subjective symptoms, QOL, and objective strength testing. Moreover, all patients reported that the program was helpful.

Our study provides a roadmap that may help address the critical lack of medical and surgical care for UI in underserved countries. Pelvic floor disorders are highly prevalent in LMIC and research shows that the prevalence of both UI and POP is higher in LMIC when compared to high-income countries (5, 15). Urinary incontinence carries a significant global impact to health and QOL and has been identified as a predictor for death when compared to continent subjects (16). Pelvic floor disorders effect significant psychological, social, and cultural impacts that are exacerbated in LMIC given lack of access to healthcare, caregiving support, and assistive products (17). Finally, UI impairs work and caretaking responsibilities, thereby causing loss of income and financial hardship (8).

PFMT is widely recognized as an effective first-line treatment for UI and is therefore an important approach in LMIC that often lack surgeons trained to provide surgical care for UI (18). In addition to significant improvements to UD symptoms and QOL, the use of PFMT can also prevent unnecessary escalation to pharmacologic and surgical treatments even when they are available. This is important given that both carry significant cost and notable adverse effects (19, 20). While surgical treatments are an important and potentially cost-effective approach to the treatment of UI, these data also support the development of combined surgical and PFMT programming as a more ideal approach to the comprehensive treatment of UI in LMIC.

Our program was carefully developed to facilitate patient comprehension and success. First, our program included not only baseline PFMT but also pelvic floor, dietary, and behavioral education. Numerous studies demonstrate the efficacy of these conservative educational and dietary approaches in the treatment of UI and underscore the importance of including these interventions along with PFMT to deliver a comprehensive pelvic floor program (21). Second, program education was delivered via comprehensive, one-on-one fashion over two sessions to help ensure patients had a thorough understanding of both the pelvic exercises and all educational materials as well. Third, program initiation included objective assessment of correct pelvic muscle activation. This assessment is important given that a significant percentage of women are unable to correctly perform pelvic muscle exercises even when reporting a prior pelvic floor knowledge or a history of performing pelvic exercises (22, 23).

The design of our program is validated by the finding that, following the 2-day training, all women reported that they understood the program and were able to demonstrate basic pelvic floor contraction. These findings are notable as multiple studies demonstrate that medical education programs are difficult in LMIC given cultural, language, and educational barriers (24, 25). Suggested approaches to optimize patient understanding and program implementation given these barriers include understanding local cultural practices and health care perceptions, use of hands on/skill-based training, and engaging local partners (25, 26).

Foremost, our study demonstrated significant improvements across a variety of subjective and objective outcomes designed to assess symptoms severity, QOL, and objective muscle strength. The use of these various measures in our study is consistent with longstanding guidelines detailing standards for research related to UI that highlight the importance of both objective and subjective measures as well as QOL assessment (27). Although success rates following PFMT for UI in high income counties (HIC) vary considerably across the literature, the improvements seen in our cohort are consistent with the large body of reported literature to date (18). We are further encouraged by our finding that success was demonstrated across our heterogeneous cohort that included women with both SUI, UUI, and MUI.

Limited experience is otherwise available to understand the feasibility and effectiveness of PFMT programming in LMIC. Randomized control trial by Kumari and colleagues demonstrated improved continence and quality of life using PFMT and behavioral therapies vs. control in rural communities in India (28). Similarly, Wagg et al. reported the successful use of a structured group-exercise pelvic floor regimen in Bangladesh, with significant decreases seen in leakage episodes (3-day bladder diary) (29). Notably, good program compliance was noted in both studies, a finding also seen in our study. This is in contrast to poor adherence rates that are commonly seen in HIC and likely related to multifactorial barriers such as work conflict, lack of transportation, or financial barriers (30). We hypothesize that the high rate of compliance observed in our study may be due patient enthusiasm for care in a community that historically has had limited or no pharmaceutical or surgical options for UI treatment.

Sustainability and scalability are critical considerations for global health programs given the need for the world health community to help address care barriers throughout LMIC. Given our encouraging experience, we are beginning the second project phase that focuses on training local providers to independently deliver this pelvic floor program to Belizean women. Such efforts are important to create sustainable access to women with UI. Similar efforts were seen in previously described study by Wagg et al., who recruited local physiotherapists and village paramedics to provide pelvic floor exercise and education interventions in Bangladesh (29). Given the lack of local physiotherapists in Belize, our research will include a diverse group of local providers (e.g., nurses, midwives, community educators) in an effort to assess whether these volunteers can effectively deliver program training.

Scalability is equally important to facilitate global impact. Program scalability is complex as numerous differences across communities, regions, and countries can impact program efficacy. The development of standardized programming and program materials is a fundamental part of scalability efforts and was carefully considered and integrated into the development of our program design and materials as detailed previously.

## Conclusions

Belizean women demonstrate good understanding of PFMT/PFE. Patients in our study also demonstrated significant improvements across a variety of subjective incontinence and quality of life outcomes, as well as objective strength testing. Study recruitment is ongoing as part of a scaled effort to develop pelvic floor services in Belize.

## Data Availability

The raw data supporting the conclusions of this article will be made available by the authors, without undue reservation.
