# Supplementary material for: Pelvic floor therapy program for the treatment of female urinary incontinence in Belize: a pilot study
Source: Front Glob Womens Health. 2024 Feb 9;5:1325259. doi: 10.3389/fgwh.2024.1325259 (PMC10884173; doi:10.3389/fgwh.2024.1325259)
Supplement: Supplementary file 1 [file Datasheet1.pdf]

How to Stay DRY

Tips to help stop leaks before they happen!

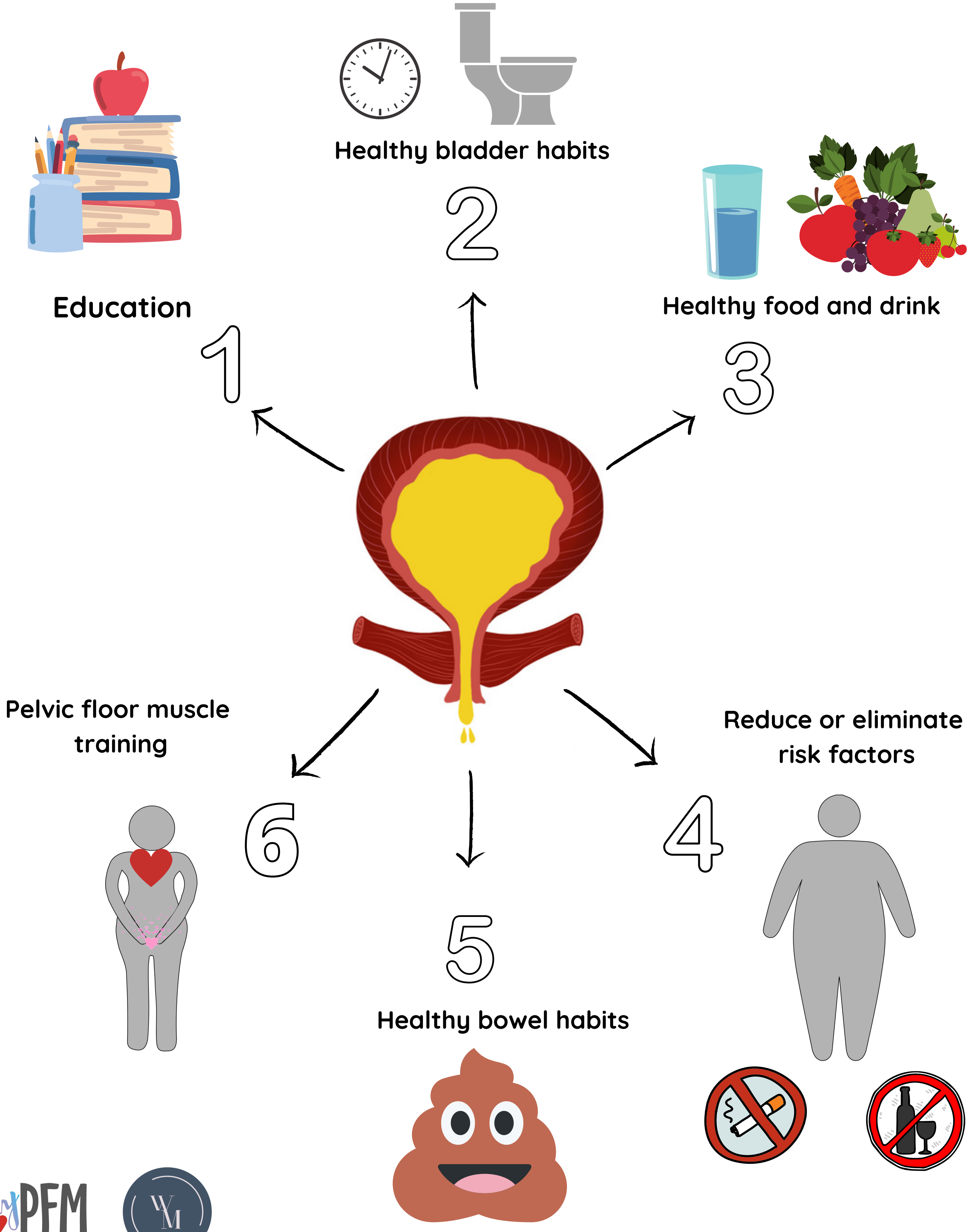

myPFM

1. EDUCATION

Location of the Pelvic Floor Muscles

Side View

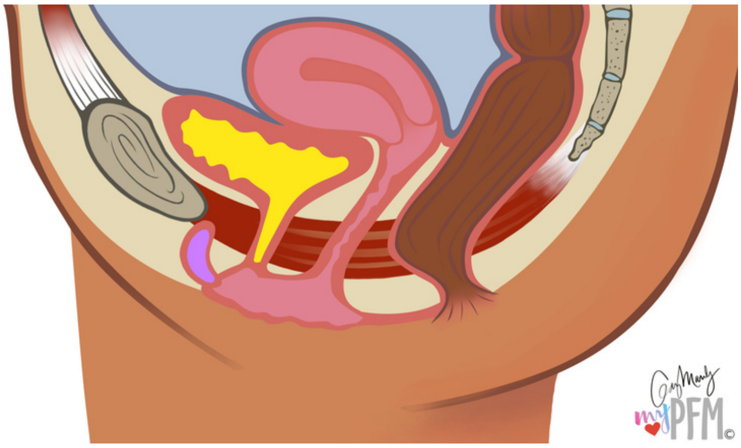

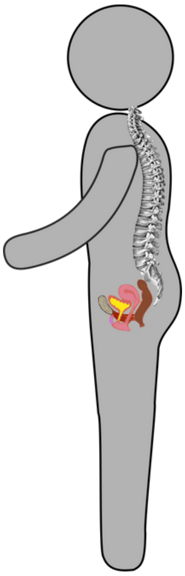

Bottom View

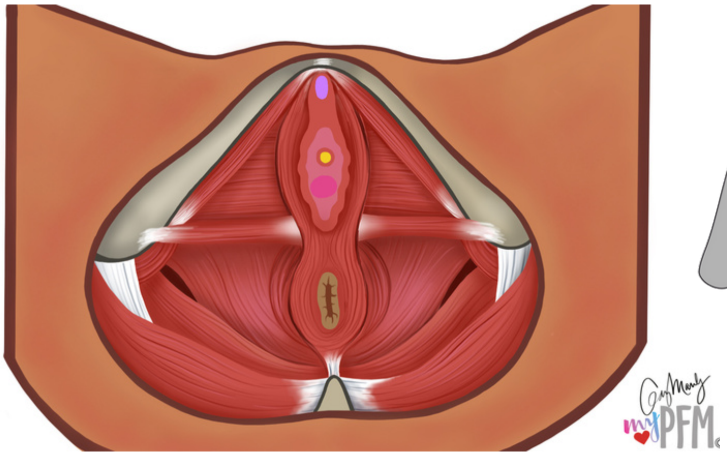

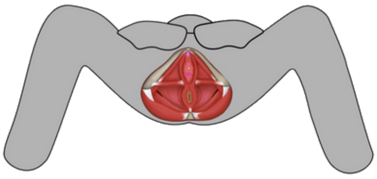

Top View

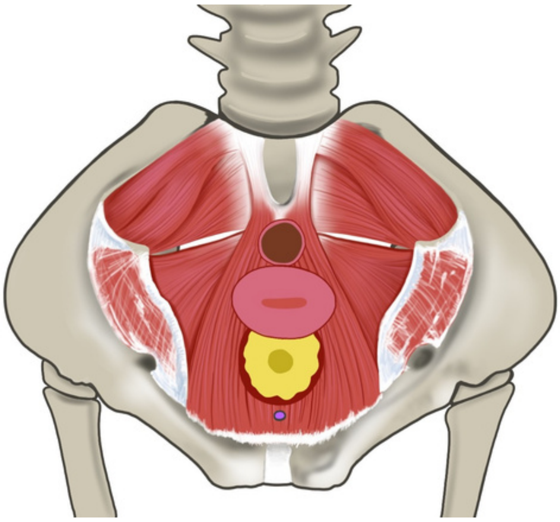

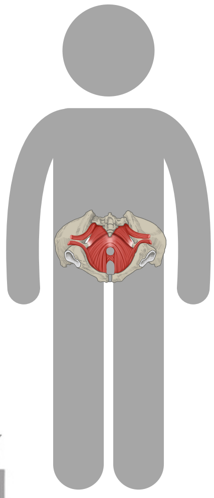

1. EDUCATION

Jobs of the Pelvic Floor Muscles

Support

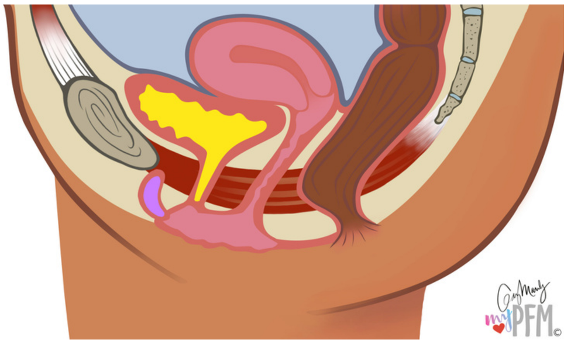

The pelvic muscles help support the organs of the pelvis like the bladder and vagina. They work kind of like a trampoline.

Sphincters

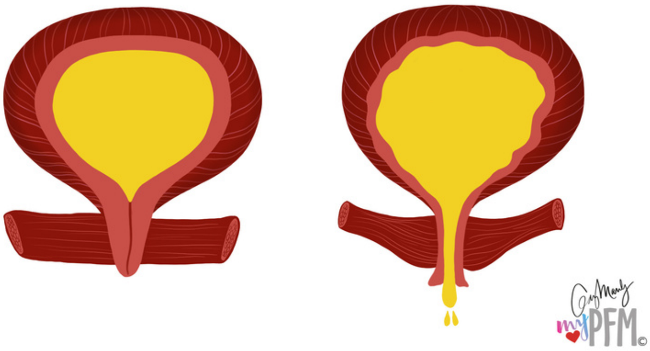

The pelvic muscles help control the bladder opening. They keep urine inside at the right times and let urine out at the right times.

Sexual

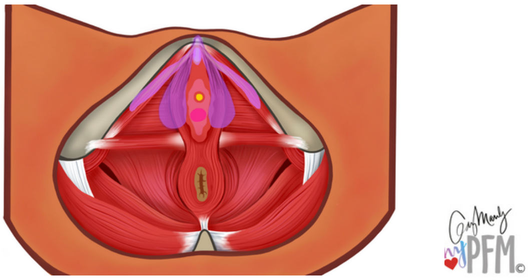

The PFM are active during sex and orgasm. They also need to relax so that sex doesn't hurt.

1. EDUCATION

When can leaks happen?

During gentle movements (standing up, rolling in bed) or other movements (lifting, jumping, exercise).

When coughing, sneezing, or laughing.

When passing gas.

During sexual activity.

When hearing running water like a faucet or shower.

When drinking caffeine or alcohol.

2. Healthy Bladder Habits

Common mistakes and how to fix them!

DON'T STRAIN. Relax your pelvic floor muscles during urination

DON'T HOLD. Listen to your bladder and urinate early when you get the urge

URINATE ON A SCHEDULE. Make time to urinate every 3-4 hours during the day

STAY HYDRATED. Drinking plenty of liquids is healthy for your bladder

URINATE BEFORE BED. Emptying your bladder before sleep can help you sleep through the night

3. Food and Drink Tips

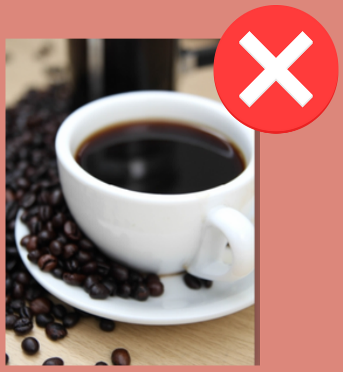

Coffee and Tea

AVOID CAFFEINE. Caffeine stimulates the bladder and causes urgency

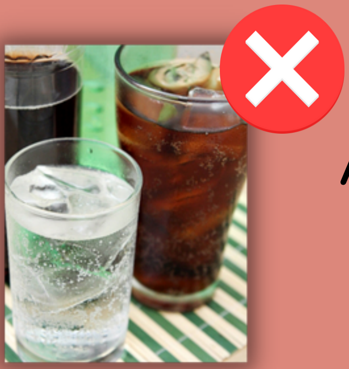

Carbonation

AVOID CARBONATION. Sodas and other carbonated drinks have lots of bladder irritants

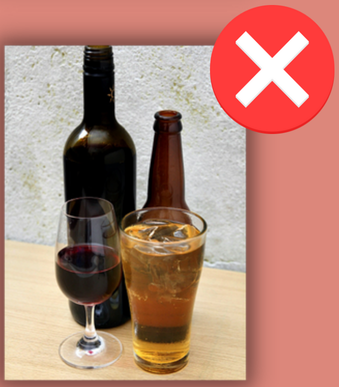

Alcohol

AVOID ALCOHOL. Alcohol stimulates the bladder and increases urine production

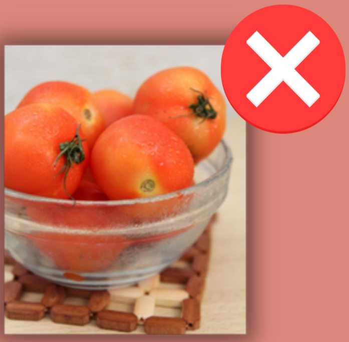

Tomatoes

AVOID TOMATOES. Tomatoes have lots of acid and can irriate the bladder

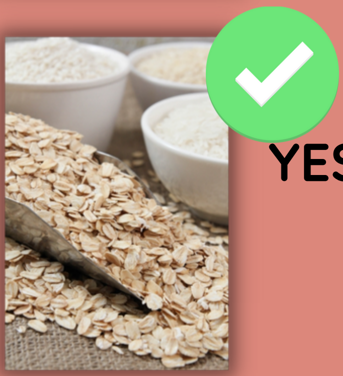

Water and Fiber

YES! Water and foods high in fiber (cereal, whole-wheat breads and pastas) are great for your bladder and bowels

4. Risk Factors

Reduce or eliminate risk factors to ↓ leaks!

RISK FACTOR

Smoking

IDEAL

Smoking doubles your risk of leaking urine

RISK FACTOR

Diabetes

IDEAL

Work with your doctor to stay in control of your sugar

RISK FACTOR

Unhealthy Weight

IDEAL

Losing just 8% of you weight can reduce your leaking by 47%!

5. Healthy Bowel Habits

Common mistakes and how to fix them!

DON'T STRAIN. Relax your pelvic floor muscles during pooping

DON'T HOLD. Listen to your bowels and go when you get the signal

TAKE ENOUGH TIME. Allow enough time to poop each day without rushing

HEALTHY FOODS AND LIQUIDS. Drinking plenty of liquids and eating fiber healthy for your bowels

USE A GENTLE SQUAT. A gentle squat forward helps poop exit easier. Avoid a straight posture.

6. Pelvic Floor Muscle Training

WHY?? Why will exercises help?

Strength

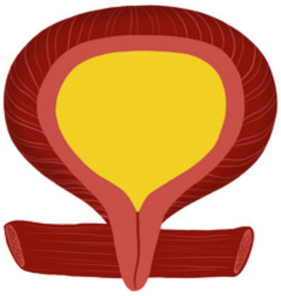

Pelvic floor exercises help strengthen the muscles around the bladder the help prevent leaking

Coordinate

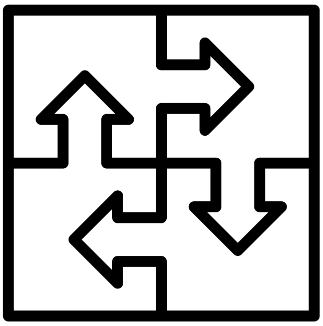

Pelvic floor exercises teach the muscles to coordinate with your bladder to relax at the right time

Support

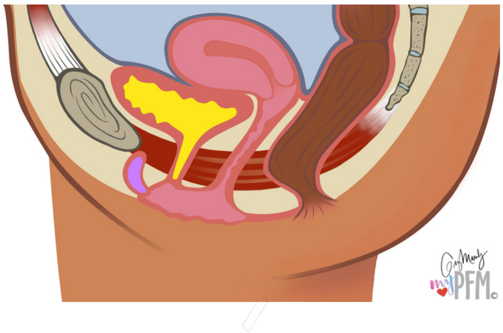

Pelvic floor exercises create strong muscles needed to support the pelvic organs

6. Pelvic Floor Muscle Training

HOW?? How do I start?

1. Identify

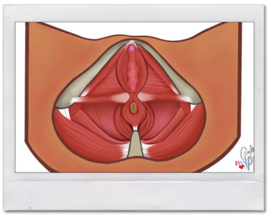

"Squeeze up and close"

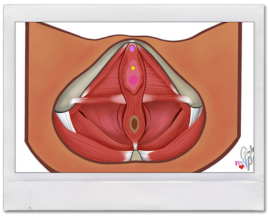

"Relax down and open"

Hint: The easiest way to identify your pelvic muscles is by practicing squeezing the anus

2. Isolate

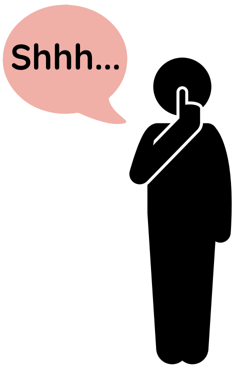

Hint: Once you can squeeze your pelvic muscles, now try to squeeze without holding your breath or squeezing other muscles like your leg or buttock muscles

3. Integrate

"Squeeze and Sneeze"

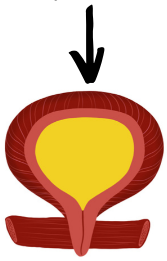

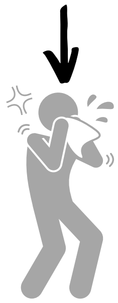

Hint: Now try to squeeze you pelvic floor muscles and then cough.

myPFM

6. Your Home Exercises

myPFM

Exercise	Position	Hold for	Repeat
**1. ENDURANCE HOLDS** (LONG HOLDS)	Lie Down	10s	10x
**2. QUICK SQUEEZES** (SHORT SQUEEZES)		2s	10x
**3. ENDURANCE HOLDS**	Sit	10s	10x
**4. QUICK SQUEEZE**		2s	10x
**5. ENDURANCE HOLDS**	Stand	10s	10x
**6. QUICK SQUEEZE**		2s	10x
**7. ACTIVITIES OF DAILY LIVING** (EXERCISES WHILE DOING NORMAL ACTIVITIES)		10s	10x
